# Taxonomic delineation of four *Zygophyllum simplex* L. (Zygophyllaceae) morphotypes using morphoanatomical, molecular (SCoT) and chemotaxonomic evidence

**DOI:** 10.1186/s12870-026-09588-2

**Published:** 2026-08-11

**Authors:** Faiza A. Shehata, Marwa M. El-Demerdash, Heba H. Elsehely, Khalil M. Saad-Allah, Dina Gad

**Affiliations:** 1 Botany and Microbiology Department, Faculty of Science, Menoufia University, Shebin El-Kom, 32511 Egypt; 2 Botany and Microbiology Department, Faculty of Science, Zagazig University, Zagazig, 44519 Egypt; 3 Botany Department, Faculty of Science, Tanta University, Tanta, 31527 Egypt; 4 Departamento de Botânica, Instituto de Biociências, Universidade de São Paulo, São Paulo, SP 05508-900 Brazil

**Keywords:** Zygophyllaceae, Micromorphology, SCoT-PCR, Infraspecific classification, Chemotaxonomic profiling

## Abstract

**Background:**

This study presents a comprehensive taxonomic adjustment of *Zygophyllum simplex* (L.) four constituent varieties in Egypt, integrating morphological, anatomical, palynological, molecular, and phytochemical analyses to evaluate their infraspecific differentiation. Samples were collected from the eastern part of Egypt and subjected to detailed morphological, anatomical, and palynological analyses with SCoT molecular genotyping and HPLC phytochemical profiling.

**Results:**

Four distinct morphological groups (informally designated as “cylindrica”, “retusa”, “orbicularis”, and “lanceolata”) were distinguished by significant differences in growth habit, leaf and stem anatomy, floral morphology, and fruit architecture. SCoT molecular marker analysis revealed a genetic similarity range of 0.825–0.881, with phylogenetic clustering strongly compatible with the morpho-anatomical groupings. HPLC profiling further identified group-specific accumulation patterns of key phenolic and flavonoid compounds, such as chlorogenic acid and catechin. While palynological analysis showed limited diagnostic value, the collective data from multiple disciplines provide robust evidence for the recognition of these four distinct morphotypes.

**Conclusions:**

Morphological, anatomical, and fruit architectural traits, together with group-specific phytochemical profiles and SCoT molecular markers, provided robust evidence for the differentiation of four distinct morphotypes within the *Z. simplex* complex. However, given the moderate resolution of SCoT markers (37.77% polymorphism), formal varietal status is not proposed at this stage. We recommend that future studies employ higher-resolution molecular approaches, such as single nucleotide polymorphism (SNP) analysis and DNA barcoding, to validate whether these morphotypes represent genetically distinct and reproductively coherent varieties. Pending such validation, these taxa should be treated as morphotypes, thereby establishing a preliminary integrative framework for their identification.

**Supplementary Information:**

The online version contains supplementary material available at 10.1186/s12870-026-09588-2.

## Background

Zygophyllaceae R.Br., a large plant family thriving in Mediterranean and semi-desert climates, encompasses a variety of trees, shrubs, and herbs adapted to arid and semi-arid regions [1]. With approximately 27 genera and 285 species, the family is classified into five subfamilies (Zygophylloideae (R. Br.) Arn., Seetzenioideae R. Br., Tribuloideae (Reichenbach) D. H. Porter, Morkillioideae (Engler) Rose & J. H. Painter, and Larreoideae Sheahan & Chase) [2]. Studies have explored the phylogenetic relationships within Zygophyllaceae, highlighting distinctions between *Zygophyllum* L. and *Tetraena* Maxim species based on morphological features like staminal appendages and fruit types [3]. Anatomical and morphological analyses play a crucial role in differentiating species within the Zygophyllaceae family [4]. A study in Saudi Arabia has introduced new subspecies combinations within the *Tetraena* Maxim. genus, emphasizing the significance of detailed morphological characteristics for species identification and differentiation [5].

*Zygophyllum simplex* (L.) distribution spans arid landscapes across the Mediterranean, Central Asia, South Africa, and Australia, where it plays a pivotal role in ecosystem dynamics [6]. The generic placement of this species remains controversial. Plants of the World Online (POWO) currently accepts *Zygophyllum simplex* L. as the accepted name, treating *Tetraena simplex* (L.) Beier & Thulin as a synonym based on the basionym *Zygophyllum simplex* [7]. In contrast, the Euro + Med PlantBase accepts *Tetraena simplex* (L.) Beier & Thulin, following the nomenclatural revision by Beier & Thulin [8], which transferred approximately 40 *Zygophyllum* species to an expanded genus *Tetraena* based on molecular phylogenetic evidence. This nomenclatural divergence stems from differing taxonomic interpretations at the genus level. Regional floras in Northeast Africa and Arabia, including the Flora of Somalia and Flora of Sudan and South Sudan, have adopted *Tetraena simplex*, reflecting continued usage of this classification in the study region [9, 10]. In the Egyptian flora, the genus *Tetraena* Maxim. is represented by four species: *Tetraena simplex* (L.) Beier & Thulin, *T. alba* (L.f.) Beier & Thulin, *T. coccinea* (L.) Beier & Thulin, and *T. aegyptia* (Hosny) Beier & Thulin [8, 11]. Previous floristic treatments across its distribution range, from North Africa through the Arabian Peninsula to South Asia, have consistently regarded *Z. simplex* as a single, polymorphic species exhibiting considerable phenotypic plasticity in response to environmental gradients.

*Zygophyllum simplex* has been traditionally employed to treat gout, edema, and asthma in Arab regions [12]. It is characterized by a diverse phytochemical profile, containing phenolic compounds, alkaloids, flavonoids, cyclic diterpenes, glycosides, and non-alkaline mineral salts [13]. This species is also utilized for treating hyperkeratotic skin conditions and is notably rich in ascorbic acid, sitosterol glucoside, phenolic acids, and an array of flavonoids, including kaempferol, quercetin, isorhamnetin derivatives, myricitrin, and luteolin-7-O-β-D-glucoside [14, 15]. The latter two compounds demonstrate significant antioxidant activity.

The Start Codon Targeted (SCoT) polymorphism is a reliable and repeatable molecular marker that is used to study genetic diversity and population structure in plants [16]. Developed based on the short-conserved region surrounding the translation start codon (ATG), SCoT markers leverage the fact that this region is highly conserved across various plant species [17]. This conservation makes SCoT markers highly transferable between different species, which is a significant advantage over other markers that are often species-specific. Its prevalence and repeatability make it a valuable tool for genetic analysis [18, 19]. Unlike other molecular markers, such as RAPD or ISSR, SCoT markers generally exhibit lower levels of interaction between genes and traits [20].

This study aims to present a comprehensive taxonomic revision of *Z. simplex* (L.) in Egypt, proposing the recognition of four distinct groups contingent upon the integrated evidence from morphoanatomical, molecular, and phytochemical analyses. Specifically, we aim: (i) to identify diagnostic morphological characters and structural adaptations that differentiate the putative taxa; (ii) to analyze phytochemical profiles as potential chemotaxonomic markers linked to stress tolerance and varietal divergence; and (iii) to assess genetic divergence and phylogenetic relationships using SCoT molecular markers. The taxonomic proposal to describe new varieties is explicitly contingent upon the outcome of these multidisciplinary analyses, and will only be advanced if significant and congruent diversification across multiple criteria is demonstrated.

## Methods

### Collection of plant samples

*Zygophyllum simplex* (L.) populations were gathered from their native environments across the eastern part of Egypt and taxonomically verified using authoritative regional floristic references [21, 22]. A total of 12 populations were sampled across four geographical regions in eastern Egypt. From each population, 10 randomly selected individuals were collected, yielding a total of 120 individuals. For morphological and anatomical analyses, 20 leaves, 20 stems, and 20 flowers were examined per individual. For palynological analysis, 30 pollen grains per individual were measured. For phytochemical profiling, leaf material from 5 individuals per population was pooled to create three replicates per population. Fresh material was collected in the field, placed in sealed plastic bags, and transported to the laboratory in an ice-cooled container within 6 h of collection. Upon arrival, specimens for anatomical analysis were fixed in FAA solution (formalin: acetic acid: 70% ethanol, 5:5:90 v/v) for 48 h, then transferred to 70% ethanol for storage at 4 °C until processing. For phytochemical analysis, leaf samples were air-dried at room temperature (25 °C) in the shade for 14 days, then ground to fine powder and stored in airtight containers at -20 °C until extraction. The formal identification of the plant material was performed by Prof. Kamal Hussien Shaltout, Professor of Plant Ecology and Taxonomy at the Botany Department, Faculty of Science, Tanta University. Voucher specimens were deposited in the publicly available Herbarium of Tanta University (TANE) under the deposition codes TANE4137 to TANE4140. These codes correspond provisionally to four morphological groups herein designated informally as the “orbicularis” type (TANE4137), the “retusa” type (TANE4138), the “lanceolata” type (TANE4139), and the “cylindrica” type (TANE4140). Formal taxonomic names are proposed only after the presentation of supporting evidence in the Results section. The phytogeographical classification of the examined samples adhered to the framework established by El-Hadidi and Hosni [23], with detailed distribution data presented in Table 1.

**Table 1:** The collection’s data of the studied *Zygophyllum simplex* groups in Egypt

var. cylindrica	S: Saint-Catherine, Sinai, 15-8-2003, F. Shehata, F. El-Shayeb & Z. Turki (TANE4140). Di: Suez; h 260 m *N* 30^o^ 06.587^\^ E. 031^o^ 40.645^\^, 3-2023, F. Shehata, Z. Turki (TANE4140 ). Nv: Wadi Degla, El Maadi, Cairo, 3-2022, F. Shehata, Z. Turki (TANE4140).
var. *lanceolata*	Di: Suez; h 260 m N 30^o^ 06.587^\^ E. 031^o^ 40.645^\^, 3-2023, F. Shehata, Z. Turki (TANE4139). Nv: Wadi Degla, El Maadi, Cairo, 3-2022, F. Shehata, Z. Turki (TANE4139 ).
var. *orbicularis*	Di: Suez; h 260 m N 30^o^ 06.587^\^ E. 031^o^ 40.645^\^, 3-2023, F. Shehata, Z. Turki (TANE4137 ).
var. *retusa*	Di: Suez; h 260 m N 30^o^ 06.587^\^ E. 031^o^ 40.645^\^, 3-2023, F. Shehata, Z. Turki (TANE4138 ).

*Abbreviations*: *TANE *Tanta University Herbarium, *Di *Isthmic desert, *Nv *Nile valley, *S *Sinai

### Macromorphological analysis

Morphological assessments were conducted on 10–15 fresh samples and supplemented by herbarium material when necessary. Observations were performed using a Leica Wild M3C stereo light microscope (Heerbrugg, Switzerland). High-resolution digital images were captured using an iPhone 13 and a Samsung A50 to document key diagnostic features.

### Palynological examination

Pollen morphology was analyzed across 60 samples, with 15 replicates representing each of the four *Z. simplex* taxa. Light microscopy facilitated preliminary observations, while scanning electron microscopy (SEM) was employed for detailed ultrastructural analysis. Pollen grains were mounted on brass stubs, sputter-coated with gold, and examined using a JEOL JSM-IT 200 Series SEM at Alexandria University. Pollen descriptions adhered to established taxonomic terminology [24–26].

### Anatomical investigation

Anatomical studies focused on vegetative structures (stems and leaves) collected from plants at comparable fruiting stages to ensure developmental uniformity. Fresh samples from the fourth internode (1–2 cm segments) were fixed in FAA (5:5:90 formalin-glacial acetic acid-70% ethanol) for 24 h. Subsequent processing included dehydration in an ethanol series, paraffin embedding, and sectioning at 10–15 μm thickness using a rotary microtome. Sections were stained with safranin and light green [27] and examined under a Zeiss stereomicroscope. Digital micrographs were obtained using an OPTIKA camera, and tissue proportions were quantified via planimetry. Anatomical terminology followed [23, 28, 29].

### SCoT PCR analysis

#### Genomic DNA extraction

Genomic DNA from the four *Z. simplex* groups was isolated from 20 mg of leaf tissue using the DNeasy Plant Mini Kit (Bio-Basic, Canada) according to the manufacturer’s protocols. The quality and concentration of the extracted DNA were assessed spectrophotometrically by determining the A_260_/A_280_ absorbance ratio using a NanoDrop UV-spectrophotometer. Samples with ratios between 1.8 and 2.0 were deemed pure and suitable for downstream applications. DNA integrity was further verified by electrophoresis on a 1% agarose gel stained with ethidium bromide, visualizing intact genomic DNA under UV light.

#### SCoT-PCR amplification

Genomic DNA was used as a template for Polymerase Chain Reaction (PCR) amplification using 12 SCoT primers in molecular assessment for samples. SCoT primers were designed from a consensus sequence derived from the previous studies by Collard and Mackill [18] and procured from Bio Basic, Canada. The sequence of the used primers is illustrated in Table 2. All SCoT primers were 18-mer and were from dataset I, which is based on highly expressed genes as described by Sawant et al. [30]. For SCoT primers design, the start codon ATG (+ 1, + 2, and + 3), ‘G’ at position + 4, ‘C’ at position + 5, and A, C, C, and A at positions + 7, +8, + 9, and + 10, respectively, were fixed (5´-----*ATG*GCTACCA—3´). PCR was carried out in a Techni TC-512 Thermal Cycler. The amplification profile comprised an initial denaturation at 94 °C for 4 min, followed by 40 cycles of denaturation at 94 °C for 1 min, annealing at 57 °C for 1 min, and extension at 72 °C for 2 min, with a final extension at 72 °C for 10 min, and the reactions were then held at 4 °C, with PCR replicates performed in triplicates.

**Table 2:** List of SCoT primers used in the study

Primer Name	Sequence (5’-3’)
SCoT 1	ACG AC ATGG CGA CCA CGC
SCoT 3	ACG AC ATGG CGA CCC ACA
SCoT 4	ACC ATG GCT ACC ACC GCA
SCoT 5	CATGG CTA CCA CTA GCG
SCoT 6	CAATGG CTA CCA CTA CAG
SCoT 7	ACA ATG GCT ACC ACT GAC
SCoT 8	ACA ATG GCT ACC ACT GAG
SCoT 10	ACA ATG GCT ACC ACC AGC
SCoT 11	ACA ATG GCT ACC ACT ACC
SCoT 12	CAA CAA TGG CTA CCA CCG
SCoT 14	ACC ATG GCT ACC AGC GCG
SCoT 15	CCA TGG CTA CCA CCG GCT

The amplified PCR products were resolved alongside a 100 bp to 1 kb DNA ladder (Bio-Basic, Canada) by electrophoresis on a 1.5% agarose gel stained with ethidium bromide. Gels were run in 1X TAE buffer at 100 V for approximately 30 min and visualized under UV light using a Bio-1D gel documentation system. Clear and reproducible DNA fragments (amplicons) were scored as present (1) or absent (0) for each primer, generating a binary data matrix. Genetic similarity between groups was estimated using the Dice similarity coefficient [30].

### UPGMA and PCA analyses of morphological and SCoT datasets

A total of 27 macro- and micromorphological characters, encompassing vegetative, floral, anatomical, and palynological traits, were scored across the four groups studied of *Z. simplex.* For quantitative characters, the Kruskal-Wallis (KW) non-parametric test was performed to assess statistically significant differences among the four groups, with H and p values reported for each character. Significant differences were considered at *p* < 0.05. Characters were coded as multistate qualitative variables (1–4), and only clearly distinguishable and consistently observed traits were retained for analysis. A data matrix was constructed from 30 individuals per variety, and phenetic similarity among groups was computed using the Jaccard similarity coefficient. A dendrogram depicting morphological relationships was generated via cluster analysis using the Unweighted Pair Group Method with Arithmetic Average (UPGMA) algorithm in PAST software (v. 4.02) [31].

For molecular analysis, polymorphic DNA fragments generated by SCoT-PCR were visually assessed, and only distinct, reproducible, and well-resolved bands were scored. Each band’s molecular weight was estimated utilizing a standard DNA ladder, and a binary matrix was constructed by scoring presence (1) or absence (0) across all samples. Genetic similarity among individuals was calculated using Jaccard’s coefficient, and a UPGMA dendrogram was generated to illustrate genetic relationships. To assess overall phenetic and genetic relationships, a combined dataset integrating macro-micromorphological characters and SCoT markers was constructed. Additionally, principal component analysis (PCA) was performed based on the Dice coefficient [32] using the SIMQUAL module, and hierarchical clustering was conducted using the UPGMA algorithm.

### HPLC profiling of phenols and flavonoids in *Zygophyllum simplex* groups

Fresh samples of *Z. simplex* were collected and transported to the laboratory in sealed plastic bags for phytochemical investigation. The aerial parts were thoroughly washed with tap water to remove surface contaminants and subsequently air-dried at ambient temperature for 4–6 weeks. The dried material was mechanically homogenized using an electrical blender to produce a fine powder (0.22 mm). The powdered plant material underwent defatting using petroleum ether to remove lipophilic compounds. For metabolite extraction, the defatted powder was subjected to maceration in 85% (v/v) aqueous methanol for 24 h with continuous agitation. The resulting mixture was centrifuged at 7000 rpm for 50 min. The supernatant was concentrated under reduced pressure, and the residual extract was reconstituted in 1 ml of methanol for chromatographic analysis. All prepared extracts were stored at 4 °C in light-protected vials and filtered through a 0.45 μm membrane before HPLC analysis.

Phytochemical characterization was performed using an Agilent 1260 series HPLC system equipped with a Zorbax Eclipse Plus C8 column (4.6 mm x 250 mm i.d., 5 μm), with three technical replicates per sample. The mobile phase consisted of a binary gradient system: (A) ultrapure water and (B) 0.05% trifluoroacetic acid in acetonitrile, delivered at a constant flow rate of 0.9 ml/min. The gradient elution program was optimized as follows: initial condition (82% A); 1–11 min (75% A); 11–18 min (60% A); 18–22 min (82% A); and 22–24 min (82% A). Column temperature was maintained at 40 °C, and phenolic compounds were monitored at 280 nm. Aliquots (5 µl) of each sample were injected in triplicate to ensure analytical reproducibility. Identification of phenolic compounds was achieved by comparing their retention times and UV-Vis spectra with those of authentic reference standards (chlorogenic acid, catechin, gallic acid, vanillic acid, and quercetin) analyzed under identical chromatographic conditions, following established protocols for phenolic compound analysis [33]. Quantification was performed using calibration curves constructed from serial dilutions of each standard.

## Results and discussion

### Macromorphological differentiation among the four groups

The four morphological groups of *Zygophyllum simplex* collected from eastern Egypt exhibited distinct and consistent macromorphological differences enabling their differentiation before formal taxonomic recognition. The plants are annual herbs exhibiting a range of growth habits from decumbent to prostrate, with coloration varying from glaucous-blue to yellowish-green. Stems are terete and bear a single longitudinal groove, with internodes measuring 0.4 to 3.5 cm in length. The leaves are succulent, simple, and sessile, displaying an oblong-oblanceolate shape with a cylindrical-flattened structure and obtuse apices, measuring 3–11 mm in length and 0.7–3 mm in width. Stipules are present and appear hyaline, ranging in shape from ovate to lanceolate.

Flowers are dark yellow and borne on very short pedicels measuring 0.2–0.5 mm in length. The calyx consists of five sepals that may be obovate, oblong, or oval in shape with obtuse to subacute apices. Petals show variation in form from orbiculate to cuneate, measuring 1.5–3 mm in length and 0.8–1.8 mm in width. The androecium comprises ten stamens, each featuring a transparent, bipartite staminal appendage, hyaline filiform filaments, and oblong-ovate anthers. The gynoecium is five-locular and ovoid in shape, with a cylindrical style that may be either curved or straight and bears a small stigma.

**Fig. 1:**
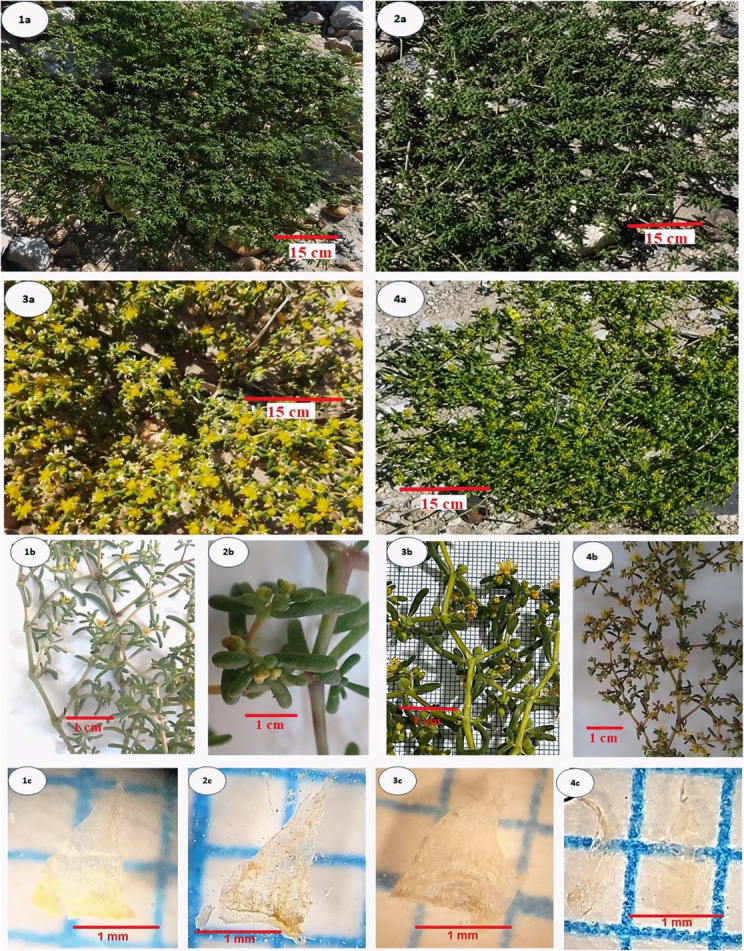
Comparative morphology of *Zygophyllum simplex* groups: (1) “cylindrica”, (2) “retusa”, (3) “orbicularis”, and (4) “lanceolata”. Panels (a) and (b) illustrate whole-plant habit characteristics, while panel (c) details stipule morphology. Scale bar is 1 mm for (c) panel

**Fig. 2:**
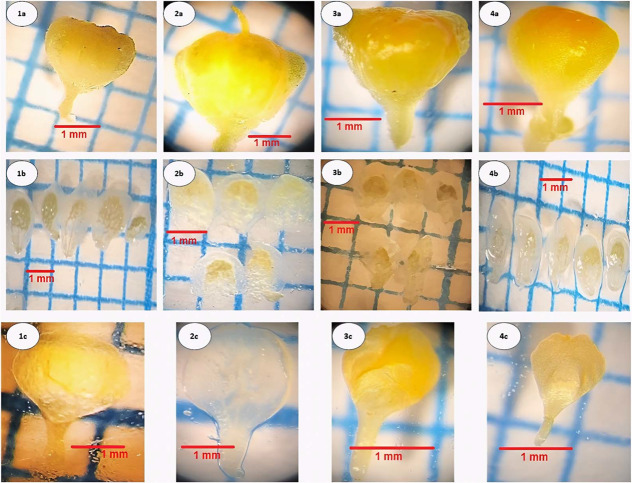
Comparative floral morphology of *Zygophyllum simplex* groups: (1) cylindrica”, (2) “retusa”, (3) “orbicularis”, and (4) “lanceolata”. Panel (a) illustrates complete floral structures, panel (b) details sepal morphology, and panel (c) highlights petal variation. Scale bars are 1 mm for all panels

**Fig. 3:**
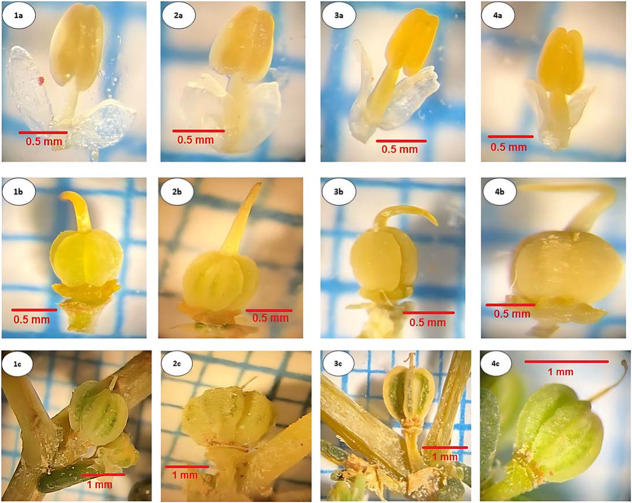
Reproductive structures of *Zygophyllum simplex* groups: (1) “cylindrica”, (2) “retusa”, (3) “orbicularis”, and (4) “lanceolata”. Panel (a) demonstrates stamen morphology, including filament and anther characteristics; panel (b) details gynoecium structure, highlighting style orientation and stigma morphology; panel (c) presents fruit architecture, emphasizing shape and surface features. Scale bars are 0.5 mm for (a) and (b), and 1 mm for (c)

Fruits are schizocarpic capsules with five-winged valves, borne on short pedicels 1–2 mm long. The capsules are ventrally curved and obovate-orbicular in outline, containing one to three seeds per locule. Seeds are dark brown to blackish-brown in color, measuring 1–1.2 mm in length and 0.4–0.6 mm in width, with a characteristically subapical hilum position. Detailed illustrations of these features are provided in Table 3; Figs. 1, 2 and 3.

**Table 3:** Morphological characters of the four studied *Zygophyllum simplex* groups (“cylindrica”, “retusa”, “orbicularis”, and “lanceolata”)

Characters /taxa		Groups			
		“cylindrica”	“retusa”	“orbicularis”	“lanceolata”
Habit		*Annual*,* decumbent*	*Annual*,* decumbent-prostrate*	*Annual*,* decumbent-prostrate*	*Annual-prostrate*
Color		Blue-green	Blue-green	Yellow-green	Yellow-green
Stem outline		Cylindrical with a groove	Cylindrical with a groove	Cylindrical with a groove	Cylindrical with a groove
Internode length (cm)		1.2–3.5	0.8–2.5	1.0-3.5	0.8–2.2
		KW: Internode length (cm): H = 1.911, *p* = 0.5911 ns			
Leaves					
Shape		*Oblong -cylindrical*	*Oblanceolate*,* flat*	*Oblanceolate*,* cylindrical*	*Oblanceolate*,* cylindrical*
Length x width (mm)		3.0-7.0 × 0.7-1.0	7.0-10.0 × 2.5-3.0	4.0-8.0 × 1.5-2.0	7.0-11.0 × 0.8-1.2
		KW: Leaf length (mm): H = 6.111, *p* = 0.1063 ns			
		KW: Leaf width (mm): H = 9.585, *p* = 0.0224^*^			
Apex		Obtuse	Obtuse	Obtuse	Obtuse
Stipules					
Shape		*Ovate*	*Ovate*	*Ovate*	*Lanceolate*
Length x width (mm)		1.7-2.0 × 0.6-1.0	1.4-1.6 × 1.3-1.5	1.8-2.1 × 0.7-1.0	1.7-1.8 × 0.4-0.6
		KW: Stipule length (mm): H = 7.992, *p* = 0.0462^*^			
		KW: Stipule width (mm): H = 9.154, *p* = 0.0273^*^			
Flowers					
Colour		Orangish yellow	Orangish yellow	Orangish yellow	Orangish yellow
Length (mm)		1.7-1.8 × 2-2.2	1.8-3.0 × 2.0-2.2	1.8-2.0 × 2.2-2.5	1.8-2.5 × 1.8-2.0
		KW: Flower length (mm): H = 5.792, *p* = 0.1222 ns			
Sepals	Shape	Obovate-oblong	Obovate- oblong	Obovate-oblong	Obovate- oblong
	Colour	green	green	green	yellowish green
	Apex	Obtuse-subacute	Obtuse-subacute	Obtuse-subacute	Obtuse-subacute
	Length x width (mm)	1.7-1.9 × 1.0-1.1	1.5-2.1 × 0.8-1.4	1.6-1.8 × 0.8-1.3	1.2-2 × 0.9-1.0
	KW: Sepal length (mm): H = 0.865, *p* = 0.8339 ns				
	KW: Sepal width (mm): H = 1.833, *p* = 0.6077 ns				
Petals	Lamina shape	*Orbicular*	*Suborbicular*	*Suborbicular*	*Cuneate*
	Apex	Obtuse	Shallow notched	Wavy	Obtuse-wavy
	Length x width (mm)	1.5-2.5 × 0.9-1.0	2.0-2.4 × 1.4-1.8	2.0-2.8 × 1.0-1.2	1.7-3.0 × 0.8-1.2
	KW: Petal length (mm): H = 1.031, *p* = 0.7938 ns				
	KW: Petal width (mm): H = 7.79, *p* = 0.0506, ns				
Androecium					
Stamen length (mm)		1.2–1.5	2.2–2.3	1.6-2.0	1.4–1.6
		KW: Stamen length (mm): H = 9.619, *p* = 0.0221^*^			
Staminal appendage	Length (mm)	0.7–1.1	1.0-1.2	0.8-1.0	0.7-1.0
	KW: Staminal appendage length (mm): H = 4.468, *p* = 0.2151 ns				
	Apex shape	*Subacute-obtuse*	*Obtuse*	*Wavy*	*Obtuse*
Anther	Shape	Ovate-oblong	Ovate-oblong	Ovate-oblong	Ovate-oblong
	Length x width (mm)	0.3-0.5 × 0.2-0.3	0.4-0.5 × 0.2-0.3	0.3-0.4 × 0.3-0.4	0.3-0.5 × 0.3-0.5
	KW: Anther length (mm): H = 2.682, *p* = 0.4434 ns				
	KW: Anther width (mm): H = 6.998, *p* = 0.072 ns				
Gynoecium	Ovary shape	Oval	Oval	Oval	Oval
	Ovary Length x width (mm)	0.9-1.2 × 0.6-0.8	1.0-1.2 × 0.6-0.8	1.7-2.2 × 1.0	0.9-1.0 × 0.8-1.0
	KW: Ovary length (mm): H = 7.903, *p* = 0.0481^*^				
	KW: Ovary width (mm): H = 8.515, *p* = 0.0365^*^				
	Style+ stigma length (mm)	*1-1.3 curved. enclosed by flower*	*1.0-1.3-straight*,* exerted*	*0.7-1.0 curved. enclosed by flower*	*0.8–1.5 curved. enclosed by flower*
Fruit					
	Pedicel length (mm)	1.4–1.6	1.4–1.8	1.4-2.0	1.0-1.5
	KW: Pedicel length (mm): H = 4.151, *p* = 0.2456 ns				
	Shape	*Obovate*	*Obovate*	*Orbiculate*	*Obovate*
	Bending downward	Bend	Bend	Bend	Bend
	Length x width (mm)	2.5-3.0 × 2.0-2.3	1.2-2.5 × 1.4-2.2	2.0-2.5 × 1.4-1.6	2.5-3.5 × 2.1-2.3
	KW: Fruit length (mm): H = 7.16, *p* = 0.067 ns				
	KW: Fruit width (mm): H = 6.258, *p* = 0.0997 ns				
	Style length (mm)	1.2–1.4	1.1–1.3	1.3–1.5	1.3–1.6
	KW: Fruit style length (mm): H = 5.833, *p* = 0.12 ns				
	Style shape	Bend to ventral side	Bend to ventral side	Bend to ventral side	Bend to ventral side

Significant differences among groups are indicated in italics. For each quantitative character, the Kruskal-Wallis (KW) test results (H and p values) are provided below the corresponding measurement row. Statistical significance is indicated as ^*^ (*p* < 0.05); ns (*p* ≥ 0.05, not significant)

The four examined groups exhibited distinct morphological characteristics that enabled their differentiation:

- “Cylindrica” group: Annual decumbent herb; blue-green coloration; oblong, cylindrical leaves; ovate stipules; orbicular petals; curved style and stigma; obovate capsules.
- “Orbicularis” group: Annual decumbent herb; yellowish-green coloration; oblanceolate leaves; ovate stipules; suborbicular petals; curved style and stigma; orbiculate capsules.
- “Retusa” group: Annual prostrate herb; blue-green coloration; oblanceolate leaves; ovate stipules; suborbicular petals with shallow notched apex; straight and exserted style and stigma; obovate capsules.
- “Lanceolata” group: Annual prostrate herb; yellowish-green coloration; oblanceolate leaves; lanceolate stipules; cuneate petals with wavy obtuse apex; curved style and stigma; obovate capsules.

These findings underscored the utility of macromorphological characters, including growth habit, leaf morphology, floral traits, and fruit shape, in distinguishing among the four studied groups.

### Palynological analysis

**Fig. 4:**
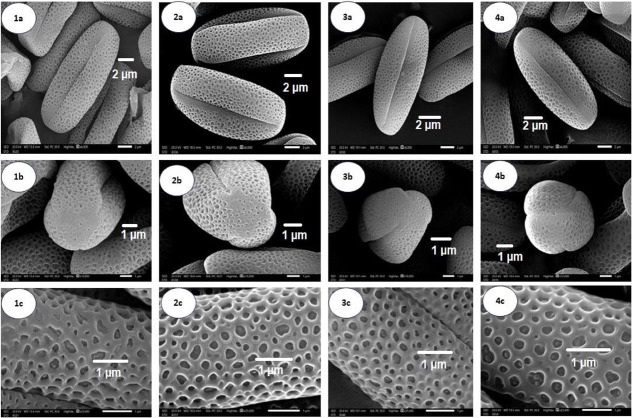
Scanning electron micrographs (SEM) of pollen grains from *Zygophyllum simplex* groups: (1) “cylindrica”, (2) “retusa”, (3) “orbicularis”, and (4) “lanceolata”. For each variety: (a) equatorial view, (b) polar view, and (c) exine ornamentation. Scale bars are 2 μm for (a), and 1 μm for (b) and (c)

Pollen grains of all four *Z. simplex* groups are monad, uniform, and trizonocolporate. Apertures number three, being long and thin with thin margins and tapering ends, measuring 10–12 μm in length. The polar/equatorial ratio ranged from 0.22 to 0.47, and pollen shape is consistently peroblate (P/E < 0.5) across all four groups, which agrees with El-Ghamery et al. [34]. Equatorial side length ranges 12.4–13.6 μm, polar side 4.5–6.4 μm, with a peroblate shape (P/E < 0.5). Exine sculpture is uniformly reticulate-perforate (holes or depressions less than 1 μm in diameter), which disagrees with Perveen and Qaiser [35]. These constant palynological characteristics are recorded in Table 4; Fig. 4.

**Table 4:** SEM of pollen grain of *Zygophyllum simplex* groups. For each quantitative character, the Kruskal-Wallis (KW) test results (H and p values) are provided below the corresponding measurement row

Characters	“cylindrica”	“retusa”	“orbicularis”	“lanceolata”
Equatorial length (µm)	13.2 × 5.0-6.0	13.6 × 5.5-6.	12.4 × 5.0-6.0	13.2 × 5.0-6.0
	KW: Equatorial length (µm): H = 11.0, *p* = 0.0117^*^			
Polar length (µm)	5.6-6.0	5.4–6.4	4.6-5.0	4.5-5.0
	KW: Polar length (µm): H = 8.401, *p* = 0.0384^*^			
Sculpture	Reticulate- perforate	Reticulate- perforate	Reticulate- perforate	Reticulate- perforate
Aperture length (µm)	11.0–12.0	10.0–11.0	11.0–12.0	11.0–12.0
	KW: Aperture length (µm): H = 5.254, *p* = 0.1541 ns			

Statistical significance is indicated as ^*^ (*p* < 0.05); ns (*p* ≥ 0.05, not significant)

The present investigation revealed no significant palynological variation among the four studied groups of *Z. simplex*. These findings align with prior research demonstrating the limited diagnostic value of pollen morphology in infraspecific differentiation [34]. The consistent failure of pollen morphology to serve as a reliable diagnostic character across multiple studies underscores the necessity of employing alternative morphological markers for accurate taxonomic classification. This pattern of conserved pollen morphology despite notable vegetative and reproductive differentiation warrants further investigation into the evolutionary and ecological factors constraining palynological diversity in these taxa. Collectively, the integrative analysis of morphological, palynological, and anatomical traits identified key diagnostic characters for morphotype delineation. These examinations provide a robust framework for species identification and differentiation within the *Z. simplex* complex.

### Micromorphological differentiation

#### Stem architectural diversity

**Fig. 5:**
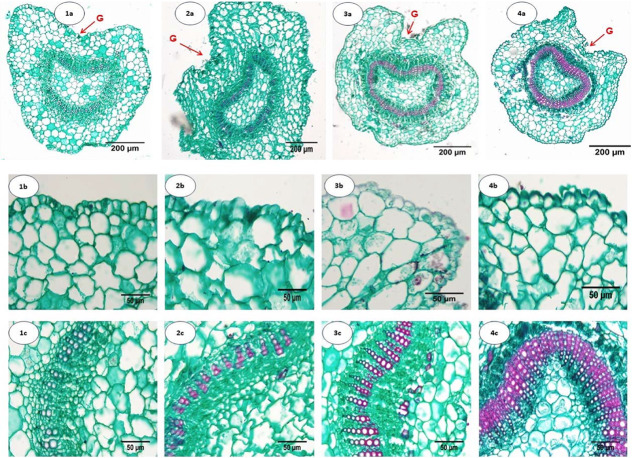
Comparative stem anatomy of *Zygophyllum simplex* groups: (1) “cylindrica”, (2) “retusa”, (3) “orbicularis”, and (4) “lanceolata”. Panel (a) whole cross-sections, (b) epidermal/cortical layers, and (c) vascular organization. Scale bars are 200 μm for (a), and 50 μm for (b) and (c). Abbreviations: groove morphology (G), and druse crystals (D)

The cross-sectional architecture of stems exhibited substantial variation among the four groups (Table 5; Fig. 5):

**Table 5:** Comparative anatomical characterization of stem and leaf morphology in *Zygophyllum simplex* groups

Characters/Taxa			Groups			
			“cylindrica”	“retusa”	“orbicularis”	“lanceolata”
Stem						
Outline			*Cylindrical*	*Cat-face*	*Cat-face*	*Cat-face*
Thickness (µm)			833.48-937.16	748.68-763.36	722.67-833.49	667.07-817.78
			KW: Stem thickness (µm): H = 5.974, *p* = 0.1129 ns			
Groove shape			*V-shape*	*U-shape*	*U-shape*	*V-shape*
Additional groove			*Absent*	*Present*	*Present*	*Absent*
Cuticle thickness (µm)			2.09–3.14	6.28–8.38	2.09–3.14	2.09–3.14
			KW: Cuticle thickness (µm): H = 6.504, *p* = 0.0895 ns			
Epidermal cell		Thickness (µm)	23.03–26.18	26.18–31.4	12.57–15.7	12.57–15.71
			KW: Epidermal cell thickness (µm): H = 9.244, *p* = 0.0262^*^			
		Cell shape	Angular-radially elongated	Angular-irregular	Isodiametric-barrel-shaped, angular	Polygonal-barrel-shaped
Cortex						
Upper parenchyma		Thickness (µm)	157.07-167.54	126.18-188.49	146.59-167.54	52.30-53.35
			KW: Upper parenchyma thickness (µm): H = 6.574, *p* = 0.0868 ns			
		Number of layers	5–6	6–7	5–6	3–4
		Cell shape	Isodiametric-irregular shape Tangentially elongated	Irregular	Isodiametric-irregular shaped radially elongated	Polygonal-irregular shaped
Crystals			*Rosette*	*Rosette*	*Rosette*	*-*
Vascular bundles						
Vascular cylinder length/width ratio			*0.88*	*0.58*	*0.75*	*0.87*
Number of vascular bundles			*26–29*	*45–48*	*51–52*	*40–47*
			KW: No. of vascular bundles: H = 9.667, *p* = 0.0216^*^			
Lateral vascular bundles			*2*	*-*	*2*	*2*
Pericyclic fibers thickness (µm)			Patches,1–2 cell Thick	-	Patches,1–3 cell thick	Patches, 2 cells thick
Phloem thickness (µm)			41.88–52.36	36.65–41.89	55.59–62.83	41.88–43.92
			KW: Phloem thickness (µm): H = 8.35, *p* = 0.0393^*^			
Xylem thickness (µm)			52.36–68.06	57.59–62.83	62.83–83.77	62.83–83.77
			KW: Xylem thickness (µm): H = 5.927, *p* = 0.1152 ns			
Number of vessels in each arch			3–6	3–5(-7)	3–6	3–8
Lower parenchyma						
Thickness(µm)			209.42–219.90	310.14–319.30	115.18-136.13	178.01-188.48
			KW: Lower parenchyma thickness (µm): H = 10.385, *p* = 0.0156^*^			
Number of layers			5–6	5–7	4–5	5–6
Cell shape			Isodiametric-irregular	Irregular	Polygonal, isodiametric, irregular	Polygonal- irregular
Pith						
Length x width (µm)			209.42-219.90 × 366.49-387.43	146.59-178.01 × 429.32-439.79	178.01-188.48 × 366.50-376.96	146.60-157.06 × 430.24-442.15
			KW: Pith length (µm): H = 9.199, *p* = 0.0268^*^			
			KW: Pith width (µm): H = 8.538, *p* = 0.0361^*^			
Number of layers			4–5	4–5	7–8	5–6
Cell shape			Isodiametric –Irregular	Isodiametric-tangentially elongated	Isodiametric-angular	Isodiametric- tangentially-radially elongated, certain cells lignified
Crystals			*-*	*Rosette*	*Rosette*	*Rosette*
Leaf						
Outline			*Cylindrical*	*Triangular-flat*	*Cylindrical with a wavy margin*	*Cylindrical with a wavy margin*
Thickness x width (µm)			707.33-716.80 × 732.98-743.46	1298.43-1319.37 × 2303.67-2324.60	732.98-753.93 × 1308.90-1329.84	1184.10-1193.72 × 1361.26-1392.67
			KW: Leaf thickness (µm): H = 10.385, *p* = 0.0156^*^			
			KW: Leaf width (µm): H = 10.385, *p* = 0.0156^*^			
Epidermis						
Cuticle thickness (µm)			2.09–3.14	2.09–3.14	2.1–3.14	2.1–3.14
			KW: Leaf cuticle thickness (µm): H = 0.431, *p* = 0.9337 ns			
Epidermal cell	Thickness (µm)		41.89–62.80	62.83–73.29	52.36–73.29	36.65–57.59
			KW: Epidermal cell thickness (µm): H = 7.166, *p* = 0.0668 ns			
	Cell shape		Polygonal-barrel-shaped, radially elongated	Irregular-barrel-shaped, tangentially elongated	Irregular, tangentially elongated	Angular-irregular, tangentially elongated
Palisade tissue						
Thickness (µm)			73.29-89.00	83.77-125.65	73.29–83.77	68.06–94.24
			KW: Palisade thickness (µm): H = 4.299, *p* = 0.2309 ns			
Upper parenchyma thickness (µm)	Thickness (µm)		115.18-136.13	260.20-262.78	136.12-146.59	397.90-418.85
			KW: Upper parenchyma thickness (µm), H = 9.974, *p* = 0.0188^*^			
	Cell shape		*Polygonal-irregular*	*Irregular*	*Irregular*	*Isodiametric-radially elongated*
Number of peripheral vascular bundles			*12–15*	*29–30*	*17–18*	*21–22*
			KW: No. periph. vascular bundles: H = 10.385, *p* = 0.0156^*^			
Central Vascular bundle	Number		1	*2 + 2 lateral*	*1*	*1*
	Thickness (µm)		62.8-78.54	68.06–78.54	83.77–94.24	90.05–94.23
			KW: Central VB thickness (µm): H = 8.504, *p* = 0.036^*^			
Lower parenchyma	Thickness (µm)		167.53–178.00	418.85–460.00	240.84–261.00	387.43–397.90
			KW: Lower parenchyma thickness (µm): H = 10.385, *p* = 0.0156^*^			
	Cell shape		*Polygonal-irregular*	*Irregular*	*Irregular*	*Isodiametric-radially elongated*
Crystals			*Rosette in parenchyma*	*Rosette in parenchyma*	*-*	*Rosette in parenchyma*,* palisade*

For each quantitative character, the Kruskal-Wallis (KW) test results (H and p values) are provided below the corresponding measurement row. Statistical significance is indicated as * (*p* < 0.05); ns (*p* ≥ 0.05, not significant)

- “Cylindrica” group: Cylindrical stem outline; U-shaped groove; angular-radially elongated epidermal cells; 3–7 layers of isodiametric-irregular upper parenchyma; 26–29 vascular bundles; vascular cylinder length/width ratio 0.88; presence of pericyclic fiber patches and lateral vascular strands; 4–5 parenchyma layers in pith; druses crystals absent in pith cells.
- “Retusa” group: Cat-face stem outline; U-shaped groove with supplementary grooves; angular-irregular epidermal cells; 3–7 layers of irregular upper parenchyma; 40–48 vascular bundles; vascular cylinder length/width ratio 0.58; absence of pericyclic fiber patches and lateral vascular strands; 4–5 parenchyma layers in pith; druses crystals present in pith cells.
- “Orbicularis” group: Cat-face stem outline; U-shaped groove with supplementary grooves; isodiametric-barrel-shaped epidermal cells; 3–7 layers of isodiametric-irregular upper parenchyma; up to 52 vascular bundles; vascular cylinder length/width ratio 0.88; presence of pericyclic fiber patches and lateral vascular strands; 7–8 parenchyma layers in pith; druses crystals present in pith cells.
- “Lanceolata” group: Cat-face stem outline; V-shaped groove without supplementary grooves; polygonal-barrel shaped epidermal cells; 3–7 layers of polygonal-irregular upper parenchyma; 40–48 vascular bundles; vascular cylinder length/width ratio 0.88; presence of pericyclic fiber patches and lateral vascular strands; 5–6 layers of lignified pith cells; druses crystals present in pith cells; druses crystals absent in upper parenchyma.

#### Leaf architectural diversity

**Fig. 6:**
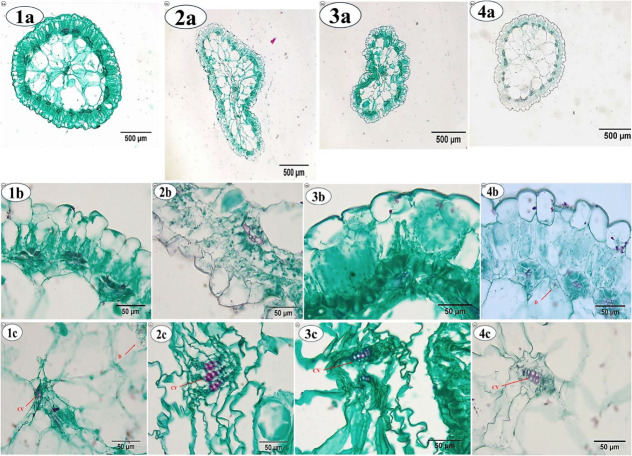
Comparative leaf anatomy of *Zygophyllum simplex* groups: (1) “cylindrica”, (2) “retusa”, (3) “orbicularis”, and (4) “lanceolata”. Panel (a) whole leaf cross-sections, (b) epidermis & palisade tissues, (c) vascular organization. Magnification: a 500 μm, b 50 μm, and c 50 μm. Abbreviations: druses (D), central vascular bundles (CV)

Cross-sectional analysis revealed distinct leaf outlines among the four groups (Table 5; Fig. 6):

- “Cylindrica” group: Cylindrical leaf outline; polygonal-barrel-shaped, radially elongated epidermal cells; single-layered palisade with Kranz anatomy; 12–15 vascular bundles; single central vascular bundle; druses crystals present in parenchyma.
- “Retusa” group: Flat triangular leaf outline; irregular-barrel-shaped, tangentially elongated epidermal cells; single-layered palisade with Kranz anatomy; 21–22 vascular bundles; two central vascular bundles plus two peripheral strands; druses crystals present in parenchyma.
- “Orbicularis” group: Cylindrical leaf outline with undulate margin; irregular, tangentially elongated epidermal cells; single-layered palisade with Kranz anatomy; 17–18 vascular bundles; single central vascular bundle; druses crystals absent.
- “Lanceolata” group: Cylindrical leaf outline with undulate margin; angular-irregular, tangentially elongated epidermal cells; single-layered palisade with Kranz anatomy; 29–30 vascular bundles; single central vascular bundle; druses crystals present in both palisade and parenchyma layers.

These micromorphological characteristics provide robust anatomical evidence supporting the distinction of four groups within *Z. simplex*.

The current anatomical findings align with the observations of Gabr and Ragab [4] in *Tetraena hamiensis*, confirming the taxonomic utility of cross-sectional outline, parenchyma stratification, vascular organization, and crystalline inclusions. These micromorphological characteristics substantiate the recognition of four distinct groups within *Z. simplex*, challenging the traditional classification in Egyptian flora [36–38] that previously recognized no infraspecific taxa.

Phylogenetic studies [8, 39, 40] robustly support the segregation of *Zygophyllum simplex* into *Tetraena simplex*, based on fruit morphology (schizocarpic vs. loculicidal capsules), staminal appendage architecture (bipartite vs. undivided), molecular phylogenetic evidence, and comparative morphological analyses. This taxonomic realignment has been subsequently adopted by many researchers [7, 12, 41], corroborating our anatomical findings. The combined evidence from macromorphological, anatomical, and palynological analyses provides compelling support for the recognition of four characteristic groups within *Z. simplex*, significantly expanding our understanding of infraspecific differentiation in this taxon. These findings underscore the importance of integrating multiple data sources for taxonomic sources, particularly in complex groups exhibiting morphological convergence. The demonstrated anatomical diversity suggests potential ecological adaptations warranting further eco-physiological investigation.

### SCoT PCR analysis

**Fig. 7:**
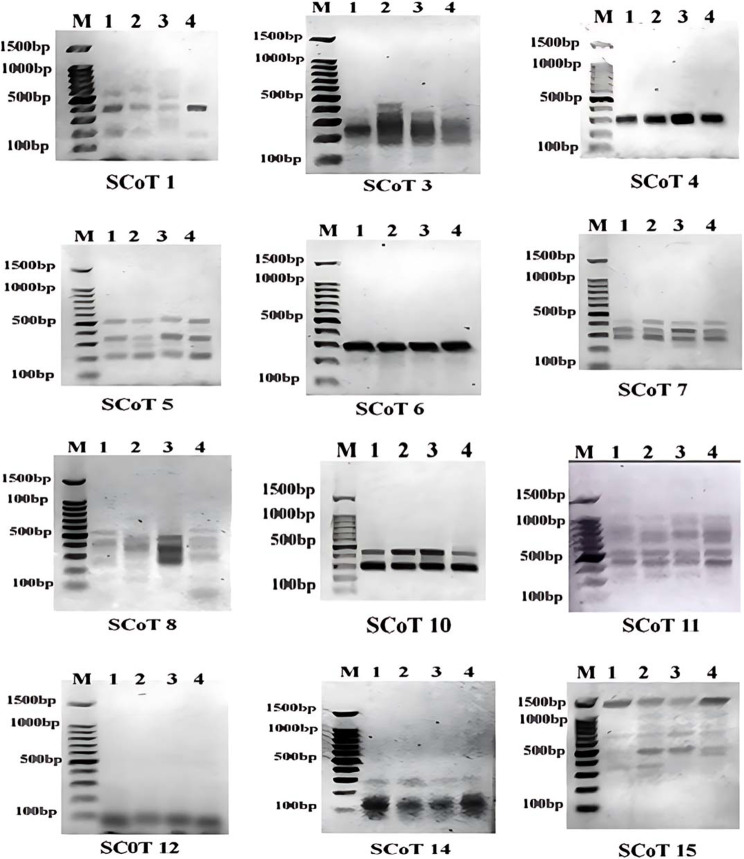
SCoT-PCR profiles generated from genomic DNA of four *Zygophyllum simplex* groups using SCoT primers 1, 3, 4, 5, 6, 7, 8, 10, 11, 12, 14, and 15. Lane M: 1 kb DNA ladder, lanes 1–4: (1) “cylindrica”, (2) “retusa”, (3) “orbicularis”, and (4) “lanceolata”, respectively

The start codon targeted (SCoT) polymorphism technique was employed to complement traditional taxonomic characterization and assess genetic diversity among the four groups. Twelve primers were selected, each generating reproducible and scorable amplification profiles. Representative profiles of the amplified fragments for each primer are depicted in Table 6; Fig. 7. Amplification with the twelve primers yielded a total of 45 apparent bands. Of these, 17 bands (37.77%) were polymorphic, demonstrating genetic variation among the four groups. The remaining 28 bands were monomorphic, indicating conserved genomic regions. For instance, primer SCoT-1 produced a total of 4 distinct and reproducible amplicons. This profile consisted of 2 monomorphic and 2 polymorphic bands, illustrating its utility in revealing both conserved and variable genetic loci.

**Table 6:** Summary of SCoT primer amplification and polymorphism analysis of four *Zygophyllum simplex* groups using SCoT primers 1, 3, 4, 5, 6, 7, 8, 10, 11, 12, 14, and 15

Primer Name	Sequences	Mo.Wt. (bp)	Total Bands	Monomorphic Bands	Polymorphic bands	Unique Bands	Polymorphism (%)
SCoT 1	ACG ACA TGG CGA CCA CGC	200–680	4	2	2	-	50.00%
SCoT 3	ACG ACA TGG CGA CCC ACA	175–435	5	1	4	-	80.00%
SCoT 4	ACC ATG GCT ACC ACC GCA	280–315	2	2	-	-	-
SCoT 5	CAA TGG CTA CCA CTA GCG	200–520	4	3	1	1	25.00%
SCoT 6	CAA TGG CTA CCA CTA CAG	380–400	2	2	-	-	-
SCoT 7	ACA ATG GCT ACC ACT GAC	280–425	3	3	-	-	-
SCoT 8	ACA ATG GCT ACC ACT GAG	160–615	7	2	5	3	71.42%
SCoT 10	ACA ATG GCT ACC ACC AGC	300–415	2	2	-	-	-
SCoT 11	ACA ATG GCT ACC ACT ACC	230–1060	7	5	2	1	28.57%
SCoT 12	CAA CAA TGG CTA CCA CCG	100	1	1	-	--	-
SCoT 14	ACC ATG GCT ACC AGC GCG	165–300	2	2	-	-	-
SCoT 15	CCA TGG CTA CCA CCG GCT	360–1500	6	3	3	2	50.00%
Total			45	28	17	7	37.77%

The overall polymorphism percentage across all primers was 37.77%. The efficiency of individual primers in detecting genetic variation differed considerably. Primer SCoT-3 exhibited the highest discriminatory power, revealing a polymorphism rate of 80.00%, followed by primer SCoT-8 (71.42%). Moderate levels of polymorphism (50.00%) were observed for primers SCoT-1 and SCoT-15. In contrast, primers SCoT-5 and SCoT-11 showed lower efficacy, with polymorphism rates of 25.00 and 28.57%, respectively (Table 6). Notably, the six primers SCoT-4, SCoT-6, SCoT-7, SCoT-10, SCoT-12, and SCoT-14 failed to yield any polymorphic bands, producing only monomorphic profiles across the tested samples.

The application of SCoT markers in this study effectively elucidated the genetic diversity and relationships among the investigated *Z. simplex* groups, providing preliminary molecular insights supporting their distinction. Its application demonstrated reproducible marker system, consistent with its growing adoption in plant phylogenetics and population genetics [18, 42, 43]. The amplification of 45 discernible bands from 12 primers confirms the reliability of the SCoT method in generating abundant genomic markers. The observed overall polymorphism of 37.77% (17 polymorphic bands) indicates a moderate level of genetic diversity within *Z. simplex* groups. This level of variation is informative for discriminatory analysis and is comparable to polymorphism rates reported in other plant species using SCoT markers, which often range from 30% to 80% depending on the genetic heterogeneity of the studied population [16, 44]. The presence of 23 monomorphic bands is equally significant, as they represent conserved genomic regions flanking start codons that are likely under functional constraint, potentially serving as valuable markers for identifying species-specific or genus-level traits [18].

A critical finding of this analysis is the considerable disparity in the discriminatory power among the 12 primers. The high polymorphism rates detected by SCoT-3 (80.00%) and SCoT-8 (71.42%) underscore their exceptional efficacy for assessing genetic variation within *Z. simplex*. Such primers are highly valuable for future diversity studies and fingerprinting of this species. Conversely, the complete lack of polymorphism from six primers (SCoT-4, -6, -7, -10, -12, and − 14) is a notable outcome. This monomorphism suggests that the genomic regions amplified by these primers are highly conserved across all accessions, possibly because they target functional genes that are critical for basic cellular processes and are therefore not subject to evolutionary divergence among these closely related taxa [45, 46]. This variation in primer performance is a well-documented characteristic of SCoT and other marker systems like ISSR and RAPD, necessitating preliminary screening to identify the most informative primers for a given taxon [47]. The primers exhibiting intermediate polymorphism rates (e.g., SCoT-1, SCoT-15 at 50.00%) offer a balanced profile, revealing both variable and conserved regions. As observed with SCoT-1, which produced two monomorphic and two polymorphic bands, such primers are particularly useful for simultaneous studies of genetic identity and differentiation. Therefore, the SCoT marker system could be considered as a useful tool for the molecular characterization of *Z. simplex* groups. The significant polymorphism revealed, particularly by primers SCoT-3 and SCoT-8, provides preliminary genetic insights supporting the proposed morphotype differentiation. These molecular findings, when integrated with the morphological, anatomical, and phytochemical data from this study, construct a powerful multidisciplinary argument for delineating the four groups. However, given the moderate overall polymorphism (37.77%) and the inherent limitations of SCoT markers for fine-scale taxonomic resolution, formal varietal recognition is not warranted at this stage. SCoT markers target conserved regions near start codons and may not capture neutral or regulatory variation necessary for robust taxonomic resolution at infraspecific level.

### Morphological and molecular clustering dendrograms and PCA

**Fig. 8:**
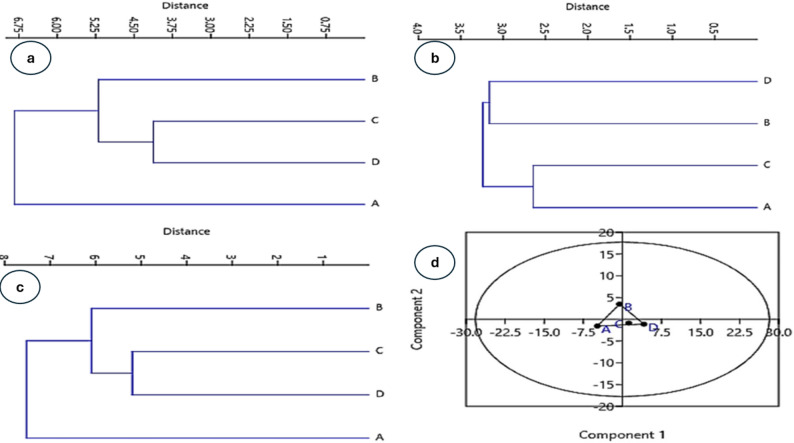
Integrative analyses of *Zygophyllum simplex* groups. **a** UPGMA dendrogram based on 27 macro and micromorphological characters. **b** UPGMA dendrogram based on SCoT molecular markers. **c** Combined UPGMA dendrogram of 75 integrated characters (morphological + SCoT). **d** PCA scatter plot of the combined dataset showing multivariate separation of the four groups (**A**, cylindrica; **B**, retusa; **C**, orbicularis; **D**, lanceolata)

The clustering analyses presented in Fig. 8 provide compelling integrative evidence supporting the distinction of four groups within the *Z. simplex* complex. Three complementary datasets, morphological (27 macro-micromorphological characters), molecular (SCoT markers), and their combination (75 characters), were employed to assess phenetic and genetic relationships among the proposed morphotypes.

The UPGMA dendrogram based on morphological characters (Fig. 8a) revealed a clear delineation of the four groups. The “cylindrica” group emerged as the most divergent, occupying a basal position. A closer phenetic affinity was observed between the “orbicularis” and “lanceolata” groups, which formed a cohesive subcluster, while the “retusa” group occupied an intermediate position. This pattern aligns with the diagnostic morphological traits documented in the morphotype characterization, particularly differences in stem outline, leaf morphology, and anatomical features such as vascular bundle arrangement and crystal distribution.

The SCoT-based dendrogram (Fig. 8b) largely corroborated the morphological findings, with the four groups forming genetically distinct clusters. The “cylindrica” group again exhibited the greatest genetic distance, reinforcing its distinctiveness. The genetic similarity coefficients (ranging from approximately 0.1 to 0.5 on the Jaccard scale) indicated moderate divergence, consistent with infraspecific variation. Notably, group *“*cylindrica” again exhibited the greatest genetic distance from the other groups, reinforcing its distinctiveness. The two groups *“*orbicularis” and *“*lanceolata” maintained their close association, while group *“*retusa” showed slightly greater affinity to this pair than suggested by morphology alone, highlighting the value of integrating multiple data sources.

The “cylindrica” group occupied the most basal and divergent position, while the “orbicularis” and “lanceolata” groups clustered together with high similarity, indicating closer affinity between these two morphotypes.

The UPGMA dendrogram generated from the combined dataset integrating 75 characters, comprising 27 macro-micromorphological traits and 48 SCoT molecular markers (Fig. 8c), provided the most comprehensive assessment of phenetic and genetic relationships among the four proposed morphotypes of *Z. simplex*. The dendrogram revealed a clear hierarchical structure with four well-defined clusters corresponding precisely to the four groups. The branching pattern demonstrated that group “cylindrica” occupies the most basal and divergent position within the tree, separating distinctly from the other three groups. This dendrogram, which is analogous to that obtained from the morphological characters, reflected that group “cylindrica” exhibited unique morphological features, including a cylindrical stem and leaf outline, V-shaped groove, and distinct vascular bundle architecture. The remaining three groups formed a larger clade that subsequently separated into two subclusters. In this dendrogram, group “retusa” branched independently, while group “orbicularis” and group “lanceolata*”* clustered together with high similarity, indicating closer phenetic and genetic affinity between these two morphotypes. This close relationship between “orbicularis” and “lanceolata” is supported by shared anatomical features, including cylindrical leaf outline with wavy margin, presence of lateral vascular bundles, and druses crystal distribution, despite differences in fruit shape and stipule morphology. This combined dendrogram demonstrated strong correspondence with both the morphology-based and SCoT-based analyses, confirming that the integration of multiple data sources yields a robust and stable differentiation framework [48]. The distinct separation of the four groups with no intermingling provided compelling evidence supporting their recognition as distinct morphotypes within the *Z. simplex* complex.

Figure 8d displays a two-dimensional principal component analysis (PCA) scatter plot derived from the same combined dataset of 75 characters, offering an orthogonal visualization of the relationships among the four groups, with minimal to no overlap. PC1, which explains the largest proportion of total variance, effectively separated group “cylindrica” from the other three groups along the horizontal axis. This separation was driven by the distinctive morphological and molecular characters that distinguish “cylindrica”, such as its unique stem outline, groove morphology, and divergent SCoT banding patterns. The second principal component (PC2) further categorized the remaining groups along the vertical axis. Group “retusa” occupied a distinct region of the ordination space, while group “orbicularis” and group “lanceolata” formed two adjacent but non-overlapping clusters, consistent with their closer relationship observed in the UPGMA dendrograms. The clear separation of all four clusters in the PCA ordination demonstrated that the combined character set captured meaningful multivariate differences that collectively distinguish each group, indicating that the groups represent discrete phenotypic and genetic entities. This multivariate separation, together with the hierarchical clustering pattern, provided robust validation for the differentiation of four distinct groups within the *Z. simplex* complex at the morphotype level.

### HPLC analysis of phenolic and flavonoid compounds

The genus *Zygophyllum* is recognized for its rich repertoire of bioactive phytochemicals, prompting this systematic investigation into the quantitative profiling of phenolic and flavonoid compounds across four distinct groups using high-performance liquid chromatography (HPLC). Notably, this study represents the first comprehensive report on the polyphenolic composition of *Z. simplex* groups, addressing a critical gap in the phytochemical literature of this medicinally significant taxon.

Our HPLC analysis (Table 7) revealed substantial intraspecific variation in flavonoid accumulation patterns among the four groups. The flavonoid catechin was exclusively detected in the “cylindrica” group (884.2 µg/g DW). Rutin exhibited maximal accumulation in the “orbicularis” group (17.0 µg/g DW), followed by the “lanceolata” group (14.0 µg/g DW). Naringenin content varied considerably, ranging from 37.7 µg/g DW in the “cylindrica” group to 287.5 µg/g DW in the “orbicularis” group. Daidzein was detected in the “retusa” group (20.8 µg/g DW) and “orbicularis” group (20.1 µg/g DW). Quercetin peaked in the “retusa” group (164.9 µg/g DW). Kaempferol was highest in the “orbicularis” group (8.8 µg/g DW). Hesperetin demonstrated exclusive presence in the “orbicularis” group (81.9 µg/g DW). Pyrocatechol, however, was found to be absent from the four investigated groups.

**Table 7:** Quantitative estimation of the polyphenol content (µg/g DW) in the aerial parts of four groups of *T. simplex* using the HPLC technique

Chemical group	Compounds	T. simplex group				LSD at 0.05	KW test	
		“cylindrica”	“retusa”	“orbicularis”	“lanceolata”		H	*p* value
Flavonoids	Catechin	884.2 ± 2.9^a^	ND^b^	ND^b^	ND^b^	2.28	10.735	0.0132^*^
	Rutin	ND^d^	4.2 ± 0.1^c^	17.0 ± 0.2^a^	14.0 ± 0.1^b^	0.74	10.532	0.0145^*^
	Naringenin	37.7 ± 0.3^d^	57.0 ± 0.2^c^	287.5 ± 1.9^a^	74.8 ± 1.1^b^	4.43	10.385	0.0156^*^
	Daidzein	8.1 ± 0.2^b^	20.8 ± 0.2^a^	20.1 ± 0.1^a^	8.0 ± 0.0^b^	0.81	9.596	0.0223^*^
	Quercetin	114.0 ± 2.3^c^	164.9 ± 1.8^a^	67.0 ± 0.9^d^	127.9 ± 2.6^b^	3.45	10.385	0.0156^*^
	Kaempferol	1.8 ± 0.0^d^	5.7 ± 0.1^b^	8.8 ± 0.1^a^	2.3 ± 0.0^c^	0.24	10.683	0.0136^*^
	Hesperetin	ND^c^	3.6 ± 0.0^b^	81.9 ± 1.8^a^	2.8 ± 0.0^b^	1.66	10.839	0.0126^*^
	Pyro catechol	ND	ND	ND	ND	0.00	-	-
Phenolics	Gallic acid	306.2 ± 3.9^b^	263.9 ± 2.4^c^	465.7 ± 3.2^a^	138.8 ± 1.5^d^	5.81	10.385	0.0156^*^
	Chlorogenic acid	171.1 ± 1.1^c^	259.3 ± 2.6^b^	1442.4 ± 8.6^a^	94.1 ± 1.2^d^	14.54	10.385	0.0156^*^
	Methyl gallate	3.3 ± 0.0^c^	4.1 ± 0.1^b^	5.6 ± 0.1^a^	3.1 ± 0.0^c^	0.37	10.683	0.0136^*^
	Caffeic acid	12.4 ± 0.2^d^	22.5 ± 0.1^b^	73.0 ± 0.4^a^	16.5 ± 0.2^c^	2.08	10.385	0.0156^*^
	Syringic acid	35.5 ± 1.1^d^	122.2 ± 3.2^b^	135.1 ± 2.4^a^	73.8 ± 1.7^c^	4.08	10.385	0.0156^*^
	Ellagic acid	4.0 ± 0.1^b^	7.0 ± 0.3^a^	4.3 ± 0.2^b^	2.2 ± 0.0^c^	0.55	10.348	0.0156^*^
	Coumaric acid	11.9 ± 0.9^c^	28.5 ± 0.3^b^	39.6 ± 0.5^a^	7.6 ± 0.0^d^	1.07	10.532	0.0145^*^
	Vanillin	29.1 ± 0.8^d^	46.9 ± 0.2^c^	173.3 ± 1.6^a^	49.2 ± 0.5^b^	2.61	10.385	0.0156^*^
	Ferulic acid	5.1 ± 0.0^c^	12.1 ± 0.3^b^	40.7 ± 0.2^a^	12.2 ± 0.1^b^	1.15	9.565	0.0227^*^
	Rosmarinic acid	14.7 ± 0.2^d^	22.2 ± 0.1^c^	49.4 ± 0.0^a^	31.6 ± 0.2^b^	0.82	10.532	0.0145^*^
	Cinnamic acid	0.4 ± 0.0^c^	0.8 ± 0.0^a^	0.6 ± 0.0^b^	0.3 ± 0.0^d^	0.04	11.000	0.0117^*^

Values are presented as mean ± standard deviation (SD). Different superscript letters within the same row indicate statistically significant differences among groups at *p* < 0.05 as determined by the Least Significant Difference (LSD) post-hoc test following a significant Kruskal-Wallis test. For each compound, the Kruskal-Wallis (KW) test results (H and p values) are provided in the last two columns

*ND *Not detected, *DW *dry weight

Statistical significance is indicated as ^*^ (*p* < 0.05); ns (*p* ≥ 0.05, not significant)

These findings align with established phytochemical reports on Zygophyllaceae, which document diverse secondary metabolites including lignans, triterpenoids, and phenolic compounds [15, 49]. The observed flavonoid patterns substantiate their role as chemotaxonomic markers and potential determinants of therapeutic efficacy, given their well-documented antioxidant, anti-inflammatory, and anticancer properties [50, 51]. The absence of pyrocatechol across all groups suggests species-specific biosynthetic pathways that require further enzymatic and genetic investigation. Collectively, our study establishes a definitive flavonoid fingerprint for *Z. simplex* groups that could be utilized as chemotaxonomic criteria for morphotype differentiation.

In addition, HPLC analysis revealed significant chemotaxonomic variation in phenolic acid profiles among the four studied *Z. simplex* groups. Chlorogenic acid (1442.4 µg/g DW) and gallic acid (465.7 µg/g DW) emerged as the dominant phenolic compounds, with the “orbicularis” group demonstrating particularly robust accumulation patterns. This group showed 3.3-fold higher gallic acid content compared to group “lanceolata” (465.6 vs. 138.8 µg/g DW) and contained 93% more chlorogenic acid than other groups. The phenolic acid distribution exhibited clear group specificity, with the “orbicularis” group producing maximal concentrations of syringic acid (135.1 µg/g DW), coumaric acid (39.6 µg/g DW), and vanillin (173.3 µg/g DW), while the “retusa” group accumulated the highest levels of ellagic acid (7.0 µg/g DW) and cinnamic acid (0.8 µg/g DW).

Furthermore, the HPLC analysis revealed prominent metabolic differences, particularly in ferulic acid content, which varied nearly 8-fold between the “orbicularis” group (40.7 µg/g DW) and “cylindrica” group (5.3 µg/g DW). Rosmarinic acid showed a 3.3-fold concentration gradient across groups, while cinnamic acid remained consistently minimal (< 1 µg/g DW) in all groups, suggesting potential rate-limiting steps in the phenylpropanoid pathway. These findings corroborate previous reports of exceptional antioxidant capacity in *Z. simplex* [52] and reflect the phylogenetic complexity observed within Zygophyllaceae, where several *Zygophyllum* taxa have been reclassified as *Tetraena* [11, 53]. The observed chemo-variation likely results from genetic determinants of biosynthetic pathways interacting with environmental adaptation pressures. Such chemical diversity carries significant implications for both morphotype identification and potential applications. The distinct phenolic profiles serve as reliable biochemical markers for morphotype authentication while explaining variability in traditional medicinal efficacy. From an industrial perspective, these results identify group “orbicularis” as the optimal source for chlorogenic acid extraction and group “retusa” for ellagic acid production. The substantial variation in bioactive compounds of *Z. simplex* groups positions it as a valuable biosource for nutraceutical development, cosmetic antioxidants, and targeted phytochemical production. Finally, to further morphotype clarification, an integrative analysis should be presented with all the characters studied in this investigation.

### Morphotype characterization of *Z. simplex* groups

The examined groups exhibited distinct morphological characteristics that enabled their differentiation. Groups *cylindrica* and *orbicularis* were presented as annual decumbent herbs, while *retusa* and *lanceolata* displayed a prostrate growth habit. Pigmentation also served as a diagnostic feature, with *cylindrica* and *retusa* exhibiting a blue-green coloration, whereas *orbicularis* and *lanceolata* appeared yellowish-green. Pending validation through higher-resolution molecular approaches (e.g., SNP analysis, DNA barcoding), these groups are provisionally designated as morphotypes. The four morphotypes can be characterized as follows:

#### *Zygophyllum simplex* (L.) morphotype ‘cylindrica’ (TANE4140)

Diagnosis: Leaf cylindrical (vs. oblanceolate); stem terete with cylindrical outline (vs. cat-face); leaf vascular bundles 12–15 (vs. 17–30); petals orbicular (vs. suborbicular or cuneate).

Morphological characterization: Annual decumbent herb, blue-green. Leaves oblong, cylindrical, 3–11 × 0.7–3 mm. Stipules ovate. Petals orbicular, 1.5–3 × 0.8–1.8 mm. Style curved. Capsules obovate. Stem terete, cylindrical outline, U-shaped groove, 26–29 vascular bundles. Leaf cylindrical, 12–15 vascular bundles, single central bundle. Druses are present in the leaf parenchyma, absent in the stem pith.

Etymology: From Latin cylindricus, referring to the cylindrical leaf and stem outline.

Distribution: Sinai and the eastern desert of Egypt.

#### *Zygophyllum simplex* (L.) morphotype ‘retusa’ (TANE4138)

Diagnosis: Leaf flat, triangular; style and stigma straight, exserted; stem without lateral vascular bundles; leaf with two central vascular bundles; petal apex shallowly notched.

Morphological characterization: Annual prostrate herb, blue-green. Leaves oblanceolate, flat. Stipules ovate. Petals suborbicular with shallowly notched apex, 1.5–3 × 0.8–1.8 mm. Style straight, stigma exserted. Capsules obovate. Stem cat-face outline, U-shaped groove with supplementary grooves, vascular bundle L/W ratio 0.58, without lateral bundles, 40–48 vascular bundles. Leaf flat, 29–30 vascular bundles, two central bundles plus two peripheral strands. Druses are present in the leaf and stem parenchyma.

Etymology: From Latin retusus, referring to the shallowly notched petal apex.

Distribution: Isthmic desert of Egypt.

#### *Zygophyllum simplex* (L.) morphotype ‘orbicularis’ (TANE4137)

Diagnosis: Fruit orbiculate; stem with two lateral vascular bundles; leaf vascular bundles 17–18; rosette crystals absent in leaf parenchyma; highest chlorogenic acid and naringenin content.

Morphological characterization: Annual decumbent herb, yellowish-green. Leaves oblanceolate, terete with wavy margin. Stipules ovate. Petals suborbicular with wavy obtuse apex. Style curved, stigma included. Capsules orbiculate. Stem cat-face outline, U-shaped groove with supplementary grooves, vascular bundle L/W ratio 0.75–0.87, with two lateral bundles, up to 52 vascular bundles. Leaf terete with wavy margin, 17–18 vascular bundles, one central bundle. Druses are absent in the leaf parenchyma, present in the stem pith.

Etymology: From Latin orbicularis, referring to the circular fruit shape.

Distribution: Isthmic desert of Egypt.

#### Zygophyllum simplex (L.) morphotype ‘lanceolata’ (TANE4139)

Diagnosis: Stipules lanceolate (vs. ovate); petals cuneate; stem with V-shaped groove, no supplementary groove; pith with 5–6 lignified cell layers; leaf vascular bundles 21–22; rosette crystals in palisade and parenchyma.

Morphological characterization: Annual prostrate herb, yellowish-green. Leaves oblanceolate, terete with wavy margin. Stipules lanceolate. Petals are cuneate with a wavy, obtuse apex. Style curved, stigma included. Capsules obovate. Stem cat-face outline, V-shaped groove without supplementary grooves, vascular bundle L/W ratio 0.75–0.87, with two lateral bundles, 40–48 vascular bundles, pith with 5–6 lignified layers. Leaf terete with wavy margin, 21–22 vascular bundles, one central bundle. Druses are present in palisade and leaf parenchyma and in stem pith; absent in upper stem parenchyma.

Etymology: From Latin lanceolatus, referring to the lanceolate stipules.

Distribution: Nile valley (Wadi Degla, Cairo) and the eastern desert of Egypt.

The following dichotomous key could be used to facilitate the differentiation of the four newly described varieties of *Z. simplex* based on macro and micromorphological characters:

1. Leaves lanate, petals orbicular, stem; terete outline, 26–29 vascular bundles, leaf; cylindrical outline, 12–15 vascular bundles………………………………………. morphotype ‘*cylindrica’.*
2. Leaves oblanceolate, petals suborbicular, cuneate, stem; cat-face outline, 40–52 vascular bundles, leaf; flat, terete outline, 40–50 vascular bundles……………………………….……………0.2.
3. Blue green plant, petals with shallow notched apex, style& stigma protruding in close flowers, stem; vascular bundle length/width ratio up to 0.58, without lateral vascular bundles, leaf; flat, 29–30 vascular bundles, 2 central vascular bundles……………………………. morphotype ‘*retusa’*.
4. Yellow green plant, petals with wavy, obtuse apex, style& stigma included in close flowers, stem; vascular bundle length/width ratio 0.75–0.87, with 2 lateral vascular bundles, leaf; terete with way margin, 17–22 vascular bundles, 1 central vascular bundle…………….………………0.3.
5. Fruit *orbiculate*, stipule ovate, petal suborbicular, stem; with u-shaped groove and additional groove, leaf; 17–18 vascular bundles, absence of rosette crystals in parenchyma tissue……………………………….……………morphotype ‘*orbicularis’.*
6. Fruit obovate, stipule lanceolate, petal cuneate, stem; with V-shaped groove and without additional groove, leaf; 21–22 vascular bundles, presence of rosette crystals in parenchyma tissue……………………………….……………morphotype *‘lanceolata’.*

While the present study provides comprehensive integrative evidence supporting the differentiation of four distinct morphotypes within *Z. simplex*, the absence of a closely related outgroup taxon limits the broader phylogenetic context for interpreting the observed variation. The inclusion of outgroup representatives, such as congeneric species (e.g., *T. alba*,* T. coccinea*) or closely related genera within Zygophyllaceae, would allow for a more robust assessment of character polarity and evolutionary directionality among the identified morphotypes. Future studies incorporating a wider taxonomic sampling, including putative ancestral or sister taxa, along with higher-resolution molecular approaches such as SNP analysis (e.g., RAD-seq, GBS) and DNA barcoding (e.g., matK, rbcL, ITS), are recommended to further elucidate the phylogenetic relationships and evolutionary processes underlying infraspecific diversification within the *Z. simplex* complex and to validate the status of these morphotypes.

## Conclusion

The multifaceted analyses performed in this study provide compelling and strong evidence for the taxonomic reclassification of *Zygophyllum simplex* into four distinct morphotypes (informally designated as *cylindrica*,* retusa*,* orbicularis*, and *lanceolata*). The congruence between macromorphological traits (growth habit, leaf shape, floral and fruit morphology), micromorphological characteristics (stem and leaf anatomical features), and molecular phylogenetics (SCoT markers) offers a powerful, multi-layered basis for this delimitation. The significant inter- group chemotaxonomic variation in phenolic and flavonoid profiles further underscores their biochemical distinctness and potential ecological or pharmacological specialization. While palynology proved to be of limited utility for differentiation at this taxonomic level, the consensus from all other datasets is unequivocal in supporting the recognition of four distinct groups.

With only 37.77% polymorphism, SCoT markers offer preliminary genetic insights but are insufficient for formal varietal recognition. Accordingly, we designate these taxa as morphotypes pending validation through higher-resolution approaches, including SNP analysis (RAD-seq, GBS) and DNA barcoding (matK, rbcL, ITS). Therefore, this work not only resolves the previously overlooked infraspecific complexity within *Z. simplex* in Egypt, but also establishes a definitive, character-rich key for their identification. These findings significantly advance the systematics of the genus *Zygophyllum* and highlight the critical importance of employing integrative approaches for resolving taxonomic complexities in closely related taxa, while also emphasizing the need for validation using higher-resolution molecular tools before formal taxonomic changes are proposed.

## Supplementary Information

Supplementary Material 1.

## Data Availability

The datasets used and/or analysed during the current study are available from the corresponding author on reasonable request.
